# Early Hypothalamic FTO Overexpression in Response to Maternal Obesity – Potential Contribution to Postweaning Hyperphagia

**DOI:** 10.1371/journal.pone.0025261

**Published:** 2011-09-28

**Authors:** Vanni Caruso, Hui Chen, Margaret J. Morris

**Affiliations:** 1 Department of Pharmacology, School of Medical Sciences, University of New South Wales, Sydney, New South Wales, Australia; 2 School of Medical and Molecular Bioscience, Faculty of Science, University of Technology, Sydney, New South Wales, Australia; University of Colorado Denver, United States of America

## Abstract

**Background:**

Intrauterine and postnatal overnutrition program hyperphagia, adiposity and glucose intolerance in offspring. Single-nucleotide polymorphisms (SNPs) of the fat mass and obesity associated (FTO) gene have been linked to increased risk of obesity. FTO is highly expressed in hypothalamic regions critical for energy balance and hyperphagic phenotypes were linked with FTO SNPs. As nutrition during fetal development can influence the expression of genes involved in metabolic function, we investigated the impact of maternal obesity on FTO.

**Methods:**

Female Sprague Dawley rats were exposed to chow or high fat diet (HFD) for 5 weeks before mating, throughout gestation and lactation. On postnatal day 1 (PND1), some litters were adjusted to 3 pups (vs. 12 control) to induce postnatal overnutrition. At PND20, rats were weaned onto chow or HFD for 15 weeks. FTO mRNA expression in the hypothalamus and liver, as well as hepatic markers of lipid metabolism were measured.

**Results:**

At weaning, hypothalamic FTO mRNA expression was increased significantly in offspring of obese mothers and FTO was correlated with both visceral and epididymal fat mass (P<0.05); body weight approached significance (P = 0.07). Hepatic FTO and Fatty Acid Synthase mRNA expression were decreased by maternal obesity. At 18 weeks, FTO mRNA expression did not differ between groups; however body weight was significantly correlated with hypothalamic FTO. Postnatal HFD feeding significantly reduced hepatic Carnitine Palmitoyltransferase-1a but did not affect the expression of other hepatic markers investigated. FTO was not affected by chronic HFD feeding.

**Significance:**

Maternal obesity significantly impacted FTO expression in both hypothalamus and liver at weaning. Early overexpression of hypothalamic FTO correlated with increased adiposity and later food intake of siblings exposed to HFD suggesting upregulation of FTO may contribute to subsequent hyperphagia, in line with some human data. No effect of maternal obesity was observed on FTO in adulthood.

## Introduction

Obesity rates are rising dramatically around the globe and its consequences represent a major public health concern [1]. Over the last 20 years, childhood obesity rates have risen greatly, such that they now constitute an ‘international epidemic of childhood obesity’ [2], [3], [4]. Obesity results from an imbalance between energy intake and energy expenditure, which is characterized by increased fat stores [5]. The development of obesity is influenced by the interaction of genetic and environmental factors [6], [7].

The intrauterine environment plays a key role and suboptimal maternal nutrition during gestation is implicated in the development of adult metabolic diseases. It is now accepted that nutritional changes during fetal development can predispose individuals to obesity and metabolic disease in adult life, resetting the expression of genes involved in energy homeostasis and consequently altering metabolic function [8], [9], [10], [11]. Intrauterine over-nutrition also predisposes the fetus to obesity and metabolic dysfunction in adulthood, and this likely contributes to the burgeoning early onset of obesity in childhood [12], [13].

Recent interest has turned to the gene-environment interactions that underlie obesity and several genes predisposing to obesity have been characterized [6], [14]. A genome-wide search for type 2 diabetes-susceptibility genes identified a common variant in the Fat mass and Obesity associated (FTO) gene on chromosome 16. Adults homozygous for the risk allele were 3 kilograms heavier and had 1.67 fold increased odds of obesity compared with those without a risk allele; this association was observed from age 7 years upwards predisposing individuals to diabetes through increased BMI [15]. Substantial increases in BMI, hip circumference and weight were confirmed [16]. Single-nucleotide polymorphisms (SNPs) in the FTO locus were associated with early-onset and severe obesity across different ethnic cohorts [17], [18], [19], [20], [21].

The hypothalamus is the main brain region regulating food intake, integrating peripheral signals such as leptin and insulin. In rodents, brain FTO levels are influenced by fasting or starvation [22], [23] and a link between FTO genotype and energy intake has been described in several human studies. The A allele was associated with increased energy intake in children [24] and adults [25]; AA homozygotes showed diminished satiety responsiveness suggesting that this common risk allele may exert at least some of its effect on appetite [26]. FTO mRNA is highly expressed in liver and brain regions critical in the control of feeding, including the arcuate (ARC), paraventricular (PVN), dorsomedial (DMN) and ventromedial (VMN) nuclei [27]. Thus FTO is well placed to play a central role in energy homeostasis and is linked to increased adiposity and energy intake. Studies at different developmental stages are required to fully understand the role of FTO in the development of obesity and the regulation of energy homeostasis.

In rats, we previously showed that intrauterine and early postnatal overnutrition altered the expression of hypothalamic appetite stimulator neuropeptide Y (NPY) and suppressor pro-opiomelanocortin (POMC) in offspring at weaning [28]. Maternal obesity promoted post weaning adiposity and glucose intolerance even when offspring consumed low-fat chow after weaning. Moreover, a significant additive impact with post weaning high fat diet (HFD) consumption was observed, markedly exaggerating metabolic disorders in offspring of obese mothers [29].

It is not known whether FTO is regulated by different levels of maternal nutrition, nor whether food intake is influenced by expression levels of FTO from an early age. To this end we examined the impact of maternal obesity and early life overnutrition induced by litter size reduction on FTO expression in the hypothalamus. As FTO has been linked to energy intake, we examined the association between hypothalamic FTO mRNA expression at weaning, and subsequent energy intake in groups of siblings weaned onto either low fat chow or HFD. As FTO expression in the liver has been linked to blood glucose levels [30], we also examined liver FTO mRNA expression in offspring from obese mothers at different pos tnatal ages along with other markers involved in lipid metabolism. Our results indicate an early impact of maternal obesity to up-regulate hypothalamic FTO, which correlated with increased subsequent intake of palatable HFD. At weaning, hepatic FTO expression was decreased by maternal obesity. No effect of maternal obesity on liver or hypothalamic FTO was observed in adult rats. Further, no effect of chronic palatable diet consumption on FTO was observed in either hypothalamus or liver.

## Materials and Methods

All animal work was approved by the Animal Care and Ethics Committee of the University of New South Wales, #06/60B.

### Maternal Obesity

**Figure 1:**
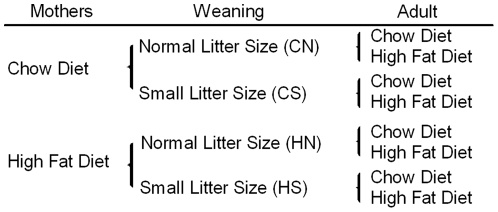
Experimental groups. Sprague Dawley dams were fed chow or HFD prior to mating and during gestation and lactation. The offspring were raised in either normal (12) or small (3) size litters. The first letter represents maternal diet and the second letter litter size. At weaning, n = 12–13 rats were sampled from CN, CS, HN and HS groups. After weaning half the remaining rats from each of these groups were fed either chow or HFD, yielding 8 groups, each comprising 10–12 rats .

Virgin female Sprague Dawley rats aged 9 weeks (Animal Resource Centre, WA, Australia) were housed at 20±2°C and maintained on a 12∶12 h light/dark cycle. Rats were assigned to two groups of equal average starting body weight and were fed either a control or a high fat diet (HFD) during gestation and lactation. Mothers in the control (n = 11) group were fed standard laboratory chow (11 kJ/g, energy 14% fat, 21% protein, 65% carbohydrate, Gordon's Specialty Stockfeeds, NSW, Australia), whereas those in the HFD (n = 9) group were fed a cafeteria diet consisting of a range of western foods (pies, cake, dim sim) supplemented with chow with added condensed milk and saturated animal fat (15.3 kJ/g, energy 34% fat, 18% protein, and 50% carbohydrate) as described previously [28], [29]. The animals used in this study were the same as those used in previous published studies [28], [29]. After 5 weeks females were mated and consumed the same diet during gestation and lactation until pups were weaned at 20 days (Fig. 1).

### Postnatal Litter size adjustment and post weaning HFD feeding

On day 1 after birth, some litters were adjusted to 3 pups (small litter), versus normal litters maintained at 12 pups, yielding 4 groups where first letter corresponds to the diet during gestation and the second letter to the litter size adjustment; (CN) chow-fed dam with normal-size litter; (CS) chow-fed dam with small-size litter; (HN) HFD-fed dam with normal-size litter; (HS) HFD-fed mum with small-size litter. At 20 days, half the male pups from each litter were weaned onto chow, while the other half were given HFD (Fig. 1).

### Sample Collection

Two time points were examined. At 20 days pups were deeply anesthetized (ketamine/xylazine 180/32 mg/kg, i.p.) prior to decapitation. At this age 12, 13, 12 and 12 pups were sampled from CN, CS, HN and HS groups respectively. At 18 weeks group sizes ranged from 10 to 12 rats. Animals were fasted overnight (14 h), then anesthetized (ketamine/xylazine 180/32 mg/kg, i.p.), naso-anal (N-A) length measured, blood was collected by cardiac puncture and rats were decapitated. Brains were rapidly removed and whole hypothalamus was dissected at 20 days while at 18 weeks the ventral hypothalamus containing ARC was dissected [29]. Livers were dissected, weighed, frozen in liquid nitrogen and stored at −80 C for subsequent RNA extraction.

### Haematoxylin and eosin staining

At 18 weeks a section of liver from each rat (n = 10–12 per group) was placed in 4% formaldehyde overnight, transferred to 70% ethanol, embedded in paraffin wax and stained for Hematoxylin and eosin. Fatty change in the liver was graded by an observer blinded to the treatment. The amount and size of lipid filled vacuoles present was graded with 0 if no evidence of lipid vacuoles; 1 few small lipid vacuoles present within hepatocytes; 2 increased number and larger lipid vacuoles within hepatocytes. The average of the grades within each treatment group was calculated.

### Quantitative Real Time PCR

RNA was extracted using Tri-reagent (Sigma, St. Louis, MO, USA) and stored at −80C (n = 6–8 per group). Spectrophotometric quantification using Biospec-nano spectrophotometer (Shimadzu Biotech, Nakagyo-ku, Kyoto, Japan) determined RNA concentration and purity. One µg of RNA was reverse transcribed simultaneously to cDNA using the Omniscript Reverse Transcription kit (Qiagen, Valencia, CA, USA) following manufacturer's instructions, and stored at −20°C. Preoptimized TaqMan probe/primers labeled with FAM (Applied Biosystems, Foster City, USA) were used for quantitative real-time PCR (Realplex 2; Eppendorf, Hamburg, Germany) on the following target genes: Fat mass and obesity associated (FTO), Rn01538187_m1; Sterol regulatory element binding protein 1c (SREBP-1C), Rn01446563_m1; Fatty acid synthase (FAS), Rn00569117_m1; Carnitine palmitoyltransferase 1a (CPT1a) Rn00580702_m1. Probes for the gene of reference (Beta Actin) were labeled with VIC. Target gene expression was quantified by single-plexing reaction and normalized by the gene of reference (beta actin). A sample from the control group was used as a calibrator. Analysis was performed using the ^ΔΔ^CT method [31].

### Statistical analysis

Statistical analysis were performed using the SPSS 17.0 Windows software (SPSS Inc., Chicago, Il) and results were expressed as mean ± SEM. Anthropomorphic, hormone and gene expression data were analyzed by one or two-way ANOVA depending on number of treatment groups, followed by a post hoc analysis using the least significant difference test (LSD) as appropriate. Liver score was analyzed by Wilcoxon matched-pairs signed rank test. Correlations between parameters measured were assessed by Pearson tests. Results were considered statistically significant when P<0.05.

## Results

### Effect of maternal and postnatal overnutrition on pups at weaning

As previously reported HFD mothers weighed 23% more than control at the time of mating and their offspring were heavier by seven days of age [28]. Pups raised in small litters had greater weight gain than those from normal size litters independent of maternal diet [28], [29]. Fat mass was increased significantly by both pre and postnatal overnutrition and leptin was higher in pups from obese mothers (HN and HS) [29].

### Liver histology

**Figure 2:**
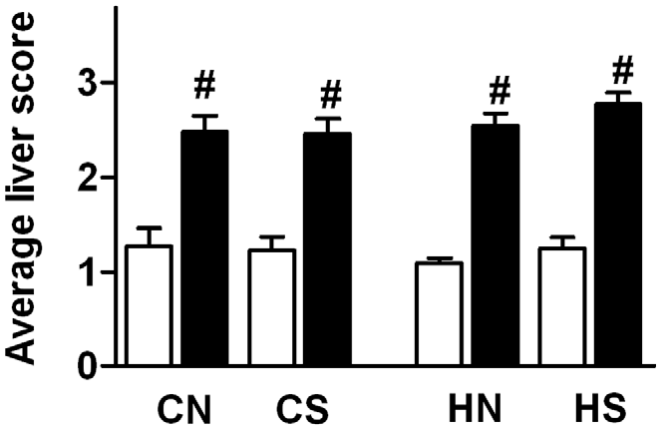
Average of liver score at 18 weeks. Liver score was analyzed by Wilcoxon matched-pairs signed rank test and results expressed as the mean ± SEM (n = 10–12 per group). CN: chow-fed mother with normal size litter. CS: chow-fed mother with small size litter. HN: HFD-fed mother with normal size litter. HS: HFD-fed mother with small size litter. Post weaning chow diet: open bars; post weaning HFD: closed bars. #, Significant post weaning diet effect: P<0.05.

At 18 weeks, scoring of liver sections revealed that post-weaning HFD increased lipid accumulation within hepatocytes in all groups independently of maternal diet and litter size adjustment (P<0.05, Fig. 2).

### Central appetite regulators at weaning and food intake of siblings

**Figure 3:**
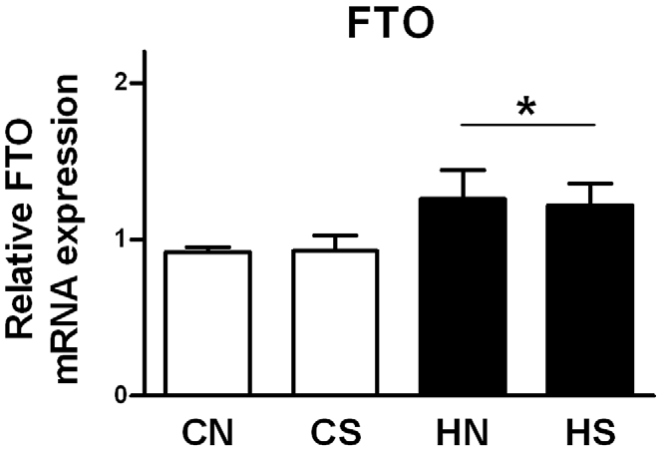
Hypothalamic FTO mRNA expression at weaning. Results are expressed as mean ± SEM (n = 6–8 per group); CN: chow-fed mother with normal size litter. CS: chow-fed mother with small size litter. HN: HFD-fed mother with normal size litter. HS: HFD-fed mother with small size litter. Data were analyzed by two-way ANOVA. *, Significant overall maternal diet effect P<0.05.

**Figure 4:**
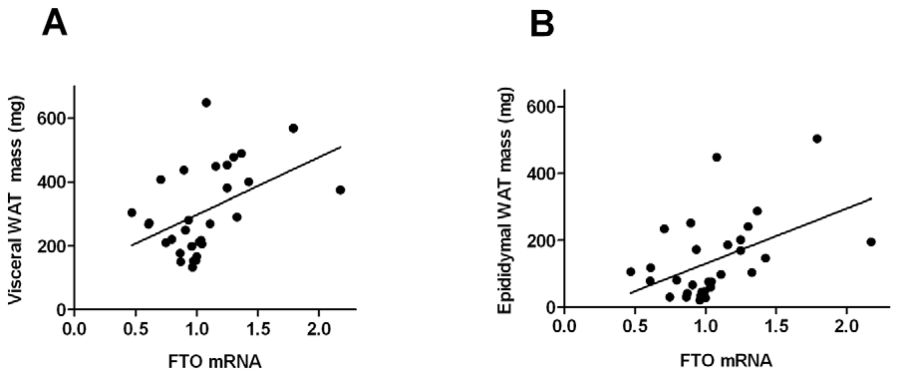
Correlation between hypothalamic FTO mRNA expression and fat mass at weaning. A) Visceral fat mass (n = 30; r = 0.459; P<0.005); B) Epididymal fat mass (n = 30; r = 0.476; P<0.005).

Hypothalamic FTO mRNA expression was increased in offspring of obese mothers (P = 0.024; n = 30; Fig. 3). FTO mRNA at weaning was significantly correlated with both visceral (P = 0.005; n = 30; r = 0.459) and epididymal (P = 0.005; n = 30; r = 0.476; Fig. 4 A and B) fat mass; the relationship with body weight approached significance (P = 0.07; n = 30; r = 0.333).

We next examined the relationship between FTO mRNA expression of rats killed at weaning and food intake of their siblings over time across each treatment group. When group means were examined, FTO mRNA expression was positively correlated with total energy intake in the four groups of adult rats fed HFD (r = 0.963; P = 0.037), but not in those fed chow. We also observed a significant correlation between FTO mRNA expression at weaning and adiposity in HFD fed siblings at 18 weeks (r = 0.96; P = 0.04). In adult rats fed HFD, Area Under the Curve (AUC) of glucose during the Glucose Tolerance Test [29] was correlated with weaning hypothalamic FTO mRNA expression of siblings from the same treatment group (r = 0.96; P = 0.04); no association was observed in those fed chow.

### Hypothalamic FTO mRNA expression in adult

**Figure 5:**
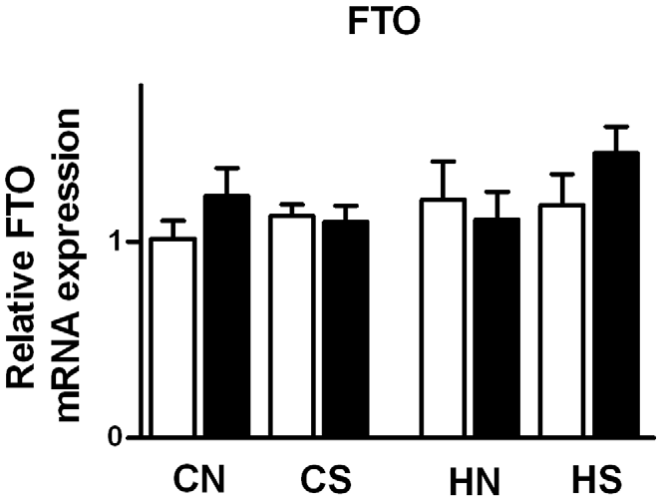
FTO mRNA expression in the ARC at 18 weeks. Results are expressed as mean ± SEM (n = 6–7) of animals consuming post weaning chow diet (open bars) and HFD (closed bars). CN: chow-fed mother with normal size litter. CS: chow-fed mother with small size litter. HN: HFD mother with normal size litter. HS: HFD mother with small size litter.

At 18 weeks, no effect of maternal diet, litter size adjustment as well as postweaning diet on FTO mRNA expression was observed (Fig. 5). However, adult body weight was correlated with hypothalamic FTO mRNA expression (r = 0.24; P = 0.05; n = 48) as well as brown adipose tissue mass (r = 0.24; P = 0.04; n = 48) and plasma insulin (r = 0.21; P = 0.01; n = 48). We also observed a correlation with hypothalamic FTO and circulating triglyceride levels (r = 0.24; P = 0.04; n = 46), but not with total fat mass (r = 0.22; P = 0.06; n = 48).

### Hepatic FTO and lipid regulators at weaning

**Figure 6:**
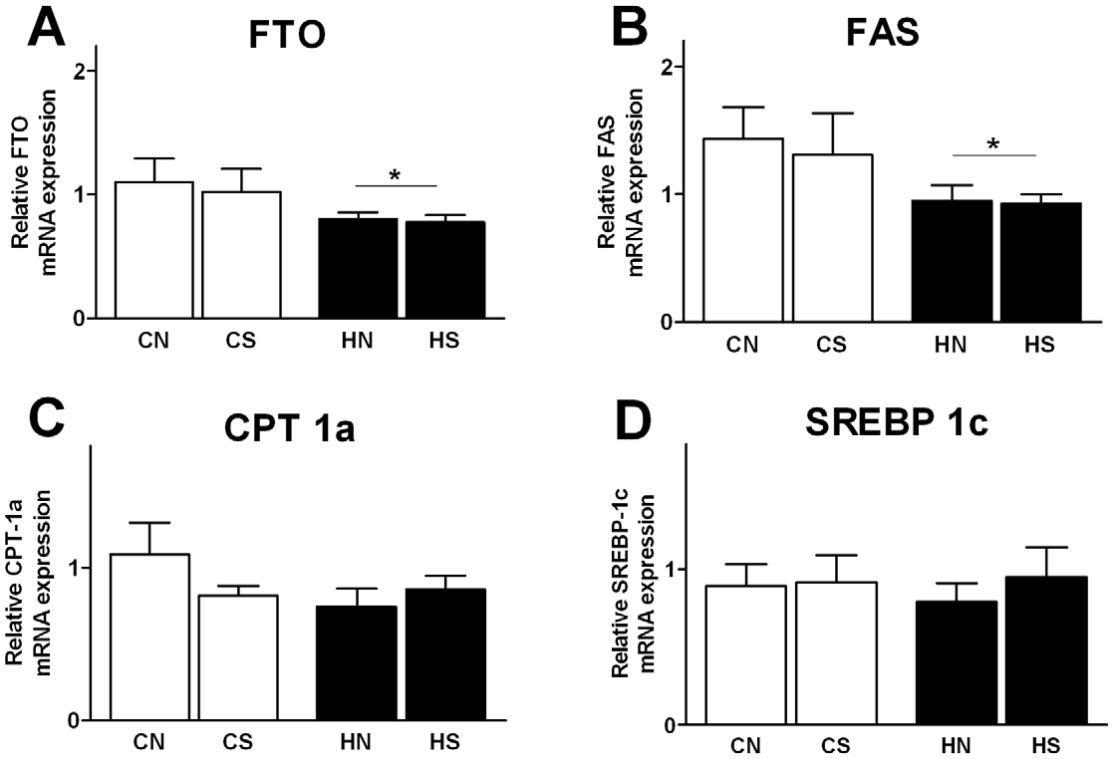
mRNA expression of hepatic markers at weaning. Results are expressed as mean ± SEM (n = 6–8). (A) FTO mRNA expression, (B) FAS, (C) CPT-1c, (D) SREBP-1c; CN: chow-fed mother with normal size litter. CS: chow-fed mother with small size litter. HN: HFD mother with normal size litter. HS: HFD mother with small size litter. Data were analyzed by two-way ANOVA.*, Significant overall maternal diet effect P<0.05.

We measured key hepatic genes involved in lipid metabolism: SREBP-1c, FAS and CPT-1a (Fig. 6). Liver FTO (P = 0.049) and FAS (P = 0.037) mRNA expression was decreased by maternal overnutrition in both HN and HS groups. CPT-1a and SREBP-1c mRNA expression remained unchanged. There was no litter size effect on the expression of hepatic markers investigated. Hepatic mRNA FTO expression was strongly correlated with the fatty acid oxidative gene CPT-1a (r = 0.62; P = 0.001; n = 33) and with the lipogenic SREBP-1c and FAS (r = 0.906; P = 0.001; n = 33). Furthermore, all hepatic markers investigated involved in lipid metabolism were significantly correlated (P<0.05).

### Hepatic FTO and lipid regulators in adult

**Figure 7:**
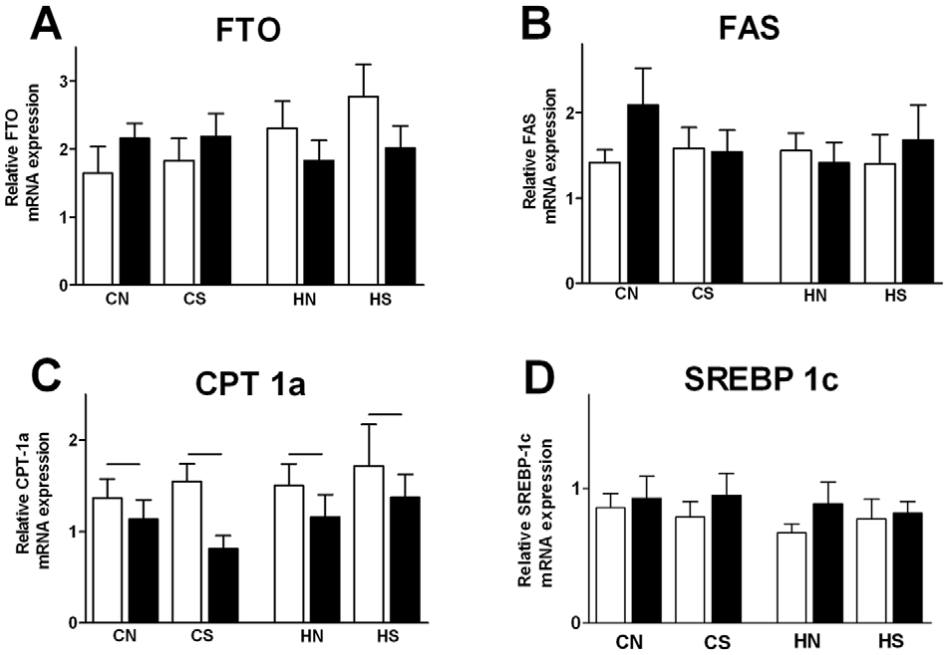
mRNA expression of hepatic markers at 18 weeks. Results are expressed as mean ± SEM (n = 6–8) of animals consuming post weaning chow diet (open bars) and HFD (closed bars). (A) FTO mRNA expression, (B) FAS, (C) CPT-1c, (D) SREBP-1c; CN: chow-fed mother with normal size litter. CS: chow-fed mother with small size litter. HN: high fat diet-fed mother with normal size litter. HS: high-fat diet-fed mother with small size litter. Bar indicates significant overall effect of postweaning diet (P = 0.014).

At 18 weeks, maternal diet and litter size adjustment as well as postnatal overnutrition did not significantly affect hepatic FTO mRNA expression (Fig. 7). FTO mRNA expression was correlated with hepatic FAS (r = 0.32; P = 0.01; n = 54) and CPT-1a (r = 0.45; P = 0.01; n = 53). Postnatal HFD reduced the expression of hepatic CPT-1a (P = 0.014; n = 54) which was negatively correlated with blood glucose (r = −0.41; P = 0.02; n = 54), fasting insulin (r = −0.32; P = 0.02; n = 52) and HOMA levels (r = −0.35; P = 0.01; n = 52). Maternal diet, litter size adjustment and postnatal overnutrition did not change the expression of the other hepatic markers involved in lipid metabolism.

## Discussion

In this study we examined the impact of maternal obesity and early life overnutrition on FTO expression in the hypothalamus and liver_ENREF_18. The novel finding of this study is that maternal obesity impacts FTO expression in young animals, with reduced impact in adulthood. Moreover, the cohort who had increased hypothalamic FTO expression at weaning consumed more energy in adulthood, but notably, this occurred only in offspring consuming palatable HFD. Modest postweaning overfeeding induced by litter size reduction had no impact on FTO, and there was no effect of HFD consumption on FTO expression.

In animals, FTO is expressed in feeding related areas and overexpression leads to increased food intake [22], [32]. We previously demonstrated that intrauterine and early postnatal overnutrition due to litter size reduction altered the expression of NPY and POMC in offspring at weaning [28], as well as their response to fasting. Our data suggest that environmental-dietary factors can also impact hypothalamic FTO expression. At weaning, hypothalamic FTO was significantly upregulated in rats from HFD mothers confirming the crucial impact of maternal diet on the programming of hypothalamic neuroendocrine circuitry. Our results suggest that overexpression of hypothalamic FTO at weaning may lead to a hyperphagic phenotype in adulthood due to differences in food intake or dietary preference, in line with other studies on human [15], [19], [24] demonstrating an association with FTO SNPs and increased intake. Increased energy intake in adulthood was seen in offspring of obese mothers, but only when rats were fed HFD [29]. This suggests that FTO may be involved in preference for palatable food, but further studies would be required to test this. Nonetheless an early increase in hypothalamic FTO mRNA expression due to maternal obesity may affect offspring energy homeostasis and their metabolism in adulthood.

Early in life hypothalamic FTO expression was correlated with fat mass, but in adulthood, the relationship between FTO and adiposity appeared to be less distinct. At 18 weeks our data did not show a significant correlation of FTO with total fat mass, in line with some human studies where the effects of FTO on body composition weakened with age [33], [34]. At this time point, maternal diet, litter size adjustment or postweaning diet did not affect the expression of FTO in the hypothalamus although it was significantly correlated with body weight, plasma insulin and triglyceride levels.

Hypothalamic FTO mRNA expression at weaning was significantly correlated with AUC of glucose of their adult siblings consuming HFD, but it is difficult to draw conclusions regarding causality. Indeed increased body weight in siblings of rats from obese mothers fed HFD may explain the reduction in glucose tolerance compared to those eating standard chow [29] as in humans the link between FTO SNPs and diabetes was abolished by BMI adjustment [15]. However, a plausible early role of FTO in the glucose metabolic pathway cannot be excluded. In humans, Hertel et al. have recently shown variants of FTO are associated with type 2 diabetes even after correction for BMI [35], although in some other studies the association is explained by the increased adiposity [36], [37]. To date, the mechanisms behind this relationship remain elusive. FTO was widely expressed in fetal brain [15] and it would also be of interest to determine whether changes in FTO expression may be present earlier in life in offspring following maternal obesity, as we have shown changes in hypothalamic appetite regulators are present by birth [38].

FTO is also expressed in metabolic tissues [30], [39], [40], however little is known concerning the role of maternal obesity and the expression of hepatic FTO. Increased metabolic disorders in childhood have been linked to maternal obesity [41] and several studies in animal models revealed that altered nutritional status during gestation and early postnatal period predisposed offspring to the development of severe hepatic steatosis [42], [43], [44], [45]. The physiological function of FTO in the liver is still unclear. Here, hepatic FTO expression was significantly reduced in offspring of obese mothers at weaning. We also investigated hepatic markers involved in lipid metabolism such as CPT-1a, SREBP-1c and FAS at two time points. The lipogenic gene SREPB-1c is normally expressed in adipocytes and involved in their differentiation [46], [47]. In mammals, SREPB-1c plays a critical role for FAS induction of lipogenesis [48], [49]. At weaning, maternal obesity was associated with downregulated hepatic FAS expression, with no change in the other hepatic markers investigated, although they were significantly correlated with FTO. Overnutrition due to litter size adjustment had no effect on hepatic FTO expression as well as that of other markers investigated. Hepatic FTO expression at weaning in rats from HFD fed mothers was significantly correlated with the fatty acid oxidative CPT-1a, SREPB-1c and FAS supporting an involvement of FTO in the regulation of hepatic lipid metabolism.

At 18 weeks, no marked changes in hepatic FTO were observed in response to any of the interventions. Few reports of physiological alterations in hepatic FTO mRNA expression exist, with one study showing fasting increased the expression of hepatic FTO in mice [30]. The precise function of FTO in the liver is not clear, and investigation of liver-specific over- and under-expression of FTO would be informative in this regard. Post-weaning HFD significantly decreased the expression of the hepatic oxidative gene CPT-1a. Decreased hepatic CPT-1a expression has been associated with reduced ability to oxidize long chain fatty acids [50], [51] although discrepancies regarding the expression of the gene CPT1-a due to nutritional state and micronutrient component of the diet have been reported [52], [53]. As we expected, histological scoring of the liver sections revealed postweaning HFD significantly exacerbated accumulation of lipid vacuoles within hepatocytes inducing steatosis.

*In summary*, our data suggest that maternal obesity may have an impact in programming the metabolic state of subsequent generations through modulation of liver and hypothalamic FTO early in life. Our findings link the deleterious impact of a suboptimal intrauterine environment induced by pre-existing maternal obesity with early increased hypothalamic FTO and subsequent development of a hyperphagic phenotype when challenged with a palatable HFD. Thus, FTO may contribute to the programming of obesity through both central and peripheral mechanisms. Additional studies are required to fully elucidate the role of FTO in liver.
